# Wartime Experiences of Single Parents by Choice

**DOI:** 10.3390/healthcare13233133

**Published:** 2025-12-02

**Authors:** Dorit Segal-Engelchin, Maya Tsfati, Alean Al-Krenawi

**Affiliations:** 1 Spitzer Department of Social Work, Ben-Gurion University of the Negev, 8410501 Beer-Sheva, Israel; 2 Resilience Research Centre, Dalhousie University, Halifax, NS B3H 4R2, Canada

**Keywords:** political violence, armed conflict exposure, single parenthood, stress, parenting experiences, coping, well-being, mental health

## Abstract

**Background/Objectives:** Despite extensive research on the outcomes faced by parents in contexts of political violence, as well as the protective factors that enhance their well-being, the experiences of single parents by choice (SPCs) in such circumstances have largely been neglected. This study sought to address this gap by examining the experiences of SPCs during the current phase of the Israel–Hamas war that began on 7 October 2023. **Method:** This qualitative study used a context-informed approach. Semi-structured interviews were conducted with 11 Israeli SPCs (5 fathers and 6 mothers), including 2 displaced due to the destruction of their homes. All participants were secular Jews, predominantly middle- to upper-middle-class, aged 40–58, and had at least one child aged 15 months to 17 years. A thematic analysis method was utilized. **Results:** Two overarching themes emerged from the interviews, shaping participants’ wartime experiences: (1) the intensified challenges associated with parenting alone in the context of armed conflict and (2) the factors that mediated the impact of these challenges. Three key challenges identified by participants included: (1) persistent perceptions of danger and threat to life; (2) heightened financial insecurity; and (3) significant disruptions to daily routines. Three systemic-level protective factors were identified as instrumental in mitigating these challenges: (1) engagement in joint familial activities; (2) the presence of a supportive work environment; and (3) social and political engagement. These factors appeared to foster resilience and enhance participants’ psychological coping capacities amidst ongoing conflict. **Conclusions:** By highlighting the distinct stressors faced by SPCs in wartime and the factors mediating their impact on well-being, our findings extend the Stress Process Model to conflict settings, enhancing understanding of how single parenting is contextually shaped during major community crises. The findings may encourage clinicians and social workers to adopt a more nuanced approach when working with parents in conflict zones, enabling them to tailor interventions to the specific needs of different family structures. For SPCs, such interventions may include tele-counseling to provide psychosocial support and guidance for parents in supporting their children, without the need for childcare or travel, as well as advocacy for workplace policies that reduce financial and emotional vulnerabilities.

## 1. Introduction

This study, grounded in the Stress Process Model (SPM), investigated the parenting experiences and mental health of Israeli single parents by choice (SPCs) during the current phase of the Israel-Hamas war that began on 7 October 2023. The SPM defines stress as a dynamic process that consists of three primary components: stressors, stress mediators, and stress outcomes [1,2,3], highlighting the mediating role of personal and social resources in shaping stress outcomes [1,2]. In this research, we show how the interplay between war-related stressors—such as exposure to violence, disrupted routines, and increased uncertainty—and the resources available to SPCs influenced not only their parenting experiences but also their emotional well-being and mental health. The results provide a nuanced understanding of the reality faced by SPCs as they navigate parenthood amid political violence, highlighting both the psychological burdens of ongoing conflict and the distinct systemic-level factors that foster resilience and coping in such high-stress environments.

### 1.1. The Effects of Exposure to Armed Conflict on the Mental Health of Parents and Children

Studies across various regions indicate that exposure to war and armed conflict negatively impacts the mental health of both adults and children, often manifesting as PTSD, anxiety, and depression [4,5]. Recent research conducted during the ongoing war in Ukraine further reinforces these findings, emphasizing the deteriorating mental health of both parents and children due to war exposure. This is demonstrated in studies on the emotional impacts of Russia’s February 2022 invasion of Ukraine, which reported a significant increase in the estimated prevalence of war-related PTSD among children of various ages [6] and revealed a strong association between war-related stressors and posttraumatic distress—including both PTSD and complex PTSD—in parents [7].

Research in other conflict zones has also revealed war-related PTSD outcomes among parents and children. For instance, a study conducted in a southern city in Israel, which was continually exposed to missile attacks during the time of the study, found high levels of posttraumatic distress among both children and mothers [8]. Another study, conducted in the Gaza Strip [9], found that 60% of parents experienced PTSD, with 42.7% reporting general mental health issues. Parents’ PTSD and anxiety were associated with similar mental health outcomes in their children. The intergenerational transmission of traumatic stress was also demonstrated in another study conducted in the Gaza Strip [5], as well as in a systematic literature review of studies conducted between 2002 and 2020 on Israeli parents and children living near the Israeli/Gaza border [10].

This evidence linking parental and children’s mental health in conflict zones underscores the importance of understanding the factors that foster parental resilience [11]. Parental resilience has indeed been associated with better mental health outcomes in children [12]. In their review of the science of resilience, Ungar and Theron [13] conclude that resilience is not merely an individual trait but emerges from access to and use of resources supporting mental health. They regard resilience as a multisystemic process in which promotive and protective factors within relational, sociocultural, and ecological systems interact to help individuals regain, maintain, or enhance well-being in the face of adversity. In line with this view, research highlights multilevel systemic factors that help parents cope with conflict-related stress and foster resilience. At the individual parent level, key factors identified include posttraumatic growth [14], activism, and civic engagement. At the family level, strong marital and family relations emerge as important facilitating factors [15]. At the community level, factors such as social support [11] and the availability of parenting interventions have been identified [16].

Despite the growing body of research on the negative outcomes faced by parents living in conflict zones and the protective factors supporting their mental health, knowledge about their personal experiences in these contexts remains limited worldwide. A recent qualitative study addressing this gap investigated Israeli parents’ personal experiences during the current Israel-Hamas war that began on 7 October 2023 [17]. The study findings indicate that while parents shared similar initial emotional responses of vulnerability and fear, their emotional experiences varied after the initial stage. However, this study included only married parents, excluding the experiences of single parents. Moreover, to the best of our knowledge, no study to date has specifically focused on the experiences of single parents living in contexts of political violence. Thus, very little is known about the impact of armed conflict exposure on the well-being of single parents and their personal experiences within these contexts. This study sought to address this gap by examining the experiences of single parents by choice (SPCs), a distinct subgroup of single parents who have actively chosen to establish a family and parent without a partner from the onset. This subgroup of SPCs is on the rise largely due to legal changes worldwide [18]. By examining the conflict-related experiences of SPCs, who are typically highly educated and economically secure [19,20], this study provides a more nuanced understanding of parenting realities in the context of political violence.

SPCs, in contrast to divorced and widowed parents, have voluntarily chosen to become parents on their own [21], thereby decoupling parenthood from couplehood [22,23,24]. Although most would have preferred to pursue parenthood with a partner, the absence of a suitable partner with whom to have children, coupled with their increasing age, led them to choose single parenthood [19,22,24,25]. Compared to divorced or widowed parents, whose transition to single parenthood entails coping with crisis and adaptation to new circumstances, SPCs undergo an organized preparation process towards single parenthood [21]. This process often includes actively creating supportive networks, restructuring their work lives, increasing economic resources, and changing their living conditions [21,22,26]. However, unlike many divorced parents, they must manage parental responsibilities, as well as financial and emotional burdens, on their own, without the support of the child’s other biological parent, both in routine times and during major crises [27,28].

The growing phenomenon of families headed by SPCs has been accompanied by research examining its implications for child adjustment and parent–child relationships. Overall, comparative studies of children raised by SPCs and those raised in heterosexual two-parent families indicate no significant differences in child development outcomes, suggesting that family processes may be more important than family structure for children’s adjustment. For example, studies comparing children of single mothers by choice with those in two-parent families have found no differences in the quality of mother–child relationships or in measures of child adjustment, including emotional and behavioral functioning [29,30]. Similarly, research comparing children of single fathers by choice with those raised in two-parent families has revealed no differences in father–child relationships, parenting quality, or children’s psychological and behavioral outcomes [31,32].

### 1.2. The Current Study

The current study focused on SPCs, both mothers and fathers, in Israel. While the phenomenon of SPCs in Israel has been consistently growing, accurate numbers remain difficult to obtain, especially for single fathers by choice, for whom data is lacking. Recent statistical data indicate a consistent rise in birth rates among never-married women over the past few decades, reflecting trends observed in other OECD countries, albeit at a slower rate. The highest rates of non-marital births are reported among women aged 35 to 39 [33], aligning with the age range at which single mothers by choice typically pursue motherhood in other countries [34].

Our study sheds light on the distinctive experiences of SPCs during the initial months of the current phase of the Israel-Hamas war, following Hamas’ attack on 7 October 2023. During the assault, widely described as among the deadliest terrorist attacks in recent decades [35], thousands of Hamas militants from Gaza breached Israel’s borders by land, sea, and air, launching rocket attacks across multiple regions [36]. The attack resulted in over 1200 deaths, mostly civilians, more than 9000 injuries, and the abduction of 240 individuals to Gaza [37]. At the same time, northern Israel came under attack from Lebanese territory. The simultaneous assaults on both the north and south borders prompted the evacuation of tens of thousands of residents from border communities [36]. The mental health consequences of the October 7 terrorist attack, whereby broad segments of Israel’s population were exposed to the traumatic impact of the attack, either directly or indirectly [38], are strongly reflected in a recent nationwide survey showing that the prevalences of probable PTSD, depression, and anxiety nearly doubled one month after the attack compared to those reported before the attack [35].

Within this context, this study sought to advance our understanding of the subjective experiences of Israeli SPCs during a period of intense armed conflict and nationwide trauma, as well as the factors shaping these experiences. The study focused on the following research questions: (1) How do SPCs in Israel experience their parenthood during the Israel-Hamas war? (2) What major resources facilitate their coping and support their well-being during the war?

## 2. Methods

To explore the experiences of SPCs during the current phase of the Israel-Hamas war that began on 7 October 2023, this qualitative study utilized a context-informed approach [39], designed to provide a holistic understanding of phenomena by examining perceptions, worldviews, and meanings shaped by institutional contexts, alongside participants’ subjective interpretations [40].

### 2.1. Participants and Recruitment

The sample consisted of 11 SPCs, 5 fathers, and 6 mothers, who were selected using purposeful, criteria-based sampling [41]. The eligibility criteria for inclusion in the study were self-identification as an SPC and having children aged 17 or younger, the age range typically used by the Israel Central Bureau of Statistics to define children. Table 1 presents key demographic characteristics of the sample. All participants were secular Jews, predominantly from middle- to upper-middle-class backgrounds, aged 40–58. They had at least one child, whose ages ranged from 15 months to 17 years. Seven participants had an academic education, two had post-high school education, and two had high school education. Nine participants resided in central Israel, and two lived in southern settlements that were severely attacked by Hamas on 7 October. These two participants were displaced due to the destruction of their homes. All fathers in our sample identified as gay men and became parents via surrogacy, while all six mothers identified as heterosexual women and became mothers via sperm donation.

Participants were recruited through snowball sampling, whereby initial participants referred eligible acquaintances to the study.

### 2.2. Data Collection

Data were collected through in-depth semi-structured interviews, using an interview guide developed by the authors (Appendix A), who have expertise in qualitative research. Participants were first invited to freely share their experiences as single parents during the Israel-Hamas war. The interview began with the question: ‘Could you please tell me about your experience as a single parent during this war?’ Participants were then invited to answer specific questions, if not already addressed, to elicit more comprehensive accounts. These questions included the following: What major challenges have you been facing as a single parent during the war? How have you been coping with these challenges? What has been helping you cope? What parenting practices have you adopted, if any, in response to these challenges? What resources have facilitated your coping during the war?

Nine interviews were conducted in November 2023, during the initial weeks of the war, a period marked by intense rocket and mortar attacks across the country. These interviews were conducted via Zoom due to the war conditions, which necessitated proximity to bomb shelters, and lasted between 45 min and an hour and a half. Two additional interviews were conducted in January 2024 via Zoom with participants who had been evacuated from their homes in southern settlements, each lasting approximately two hours. The interviews were conducted until thematic saturation was reached, at which point no new codes or categories emerged during the analysis [42]. All interviews, conducted by the second author, were recorded with participants’ permission and fully transcribed. Participants were also asked to complete a brief demographic questionnaire via email.

### 2.3. Data Analysis and Trustworthiness

Data analysis was carried out utilizing the six-stage thematic analysis method established by Braun and Clarke [42]. In Stage 1, the researchers immersed themselves in the data through multiple readings of the interview transcripts. Stage 2 involved open coding, allowing for the identification of fundamental units of meaning. During Stage 3, the initial codes were organized into potential themes, and relevant data excerpts were assigned to each theme. Stage 4 involved reviewing the proposed themes in relation to both the coded extracts and the entire data set. In Stage 5, each final theme was clearly defined and named. Ultimately, an analysis report was compiled, highlighting compelling data excerpts that were later presented in the Results section.

The data were analyzed by the first and second authors, both independently and collaboratively using a codebook. They first conducted separate coding and then compared their findings, refining codes as needed. This method, known as investigator triangulation [41], helped address selective perception and interpretive bias, thereby enhancing the trustworthiness of the results. Any discrepancies that arose during coding were discussed until consensus was reached. A sample coding memo is presented in Table 2. Trustworthiness was additionally strengthened through reflexivity [43,44].

### 2.4. Reflexivity

The authors reflected on the potential impact of their own experiences—having been exposed to the same war circumstances as the participants, a phenomenon described as shared traumatic reality [45]—on the research. This reflection enhanced their ability to maintain objectivity as observers and listeners, addressing a major challenge inherent in research involving shared trauma [46]. The second author’s positionality as a single mother by choice (SMC) was particularly noteworthy, given her role as the interviewer. Her lived experience as an SMC enabled her to recognize and identify with participants’ heightened sense of vulnerability, as well as with the varied stressors associated with single parenting described in the interviews. The mutual and simultaneous experience of the war within the same sociocultural context and social positioning as SPCs led to a partial blurring of boundaries between the second author and participants, fostering increased openness in participants’ accounts during the interviews and thereby providing the first author with deeper access to their experiences.

Nevertheless, her social positioning as an SPC also had the potential to introduce bias. To address this, several strategies were employed, including maintaining a reflexive journal throughout the research process, in which she recorded her feelings and thoughts about the data that emerged in the interviews and the themes identified during analysis. Additionally, she routinely shared her personal reflections with the co-authors for critical discussion, which supported a more reflexive stance and helped differentiate her own experiences and challenges from those of the participants. Finally, analytic decisions were systematically documented through an audit trail, enhancing transparency and reducing the potential influence of personal bias. These strategies are consistent with practical recommendations for researchers working within shared traumatic realities to address the ethical challenges inherent in such contexts. The recommendations highlight, among other considerations, the need for researchers to recognize that experiencing the same threat as their participants may constrain their professional functioning, to cultivate high levels of self-awareness and insight into their anxiety responses, and to establish supportive professional and personal frameworks [46].

### 2.5. Ethical Considerations

Ethical approval for this study was granted by the Ethics Committee of Ben-Gurion University of the Negev (Approval Number: OG01112023). The research was conducted by established ethical standards, including the principles of informed consent and confidentiality. Before the commencement of interviews, participants were briefed on the study’s objectives and procedures and then provided their informed consent by signing a form. They were also assured that their identities would be kept confidential in any future publications, with pseudonyms utilized to safeguard the confidentiality of the data collected. Consequently, all names referenced in this study are pseudonyms.

## 3. Results

Analysis of the interviews revealed two key themes that shaped participants’ experiences during the war: the exacerbated challenges they faced as single parents and the factors that helped them cope with these challenges. Table 3 summarizes the themes, subthemes, and operational definitions, reflecting participants’ experiences as described in their interviews.

### 3.1. Challenges Encountered by SPCs During the War

Participants’ accounts revealed three specific challenges encountered during the Israel-Hamas war: (1) a sense of danger and threat to life; (2) financial insecurity; and (3) disruption to daily life. All participants emphasized that these hardships were exacerbated by their status as single parents.

### 3.2. Sense of Danger and Threat to Life

All participants initially discussed their general experiences during the first days of the war, and then shifted to detailing their specific war-related experiences as single parents. They reported feeling extremely fearful due to the immediate threats they faced, emphasizing that the fear of death was central to their overall experience. The extreme fear is reflected in the words of a mother of an eight-year-old son and a six-year-old daughter, living in a southern settlement that was invaded by Hamas on 7 October.

*I told her [her daughter] that if the terrorists would enter our house we should hide under the bed, and that’s what we actually did, when we heard them in our house, shouting, slamming doors, and breaking things … We stayed under the bed for eight hours!! These were hours of fear, of threat, a dire threat to our lives*!

(P1)

The relentless rocket sirens and missile attacks across the country led all participants, regardless of their region of residence, to feel threatened, both for their own lives and for the lives of their children. This is illustrated by the following words of a mother of a sixteen-year-old daughter living in central Israel.

*During the first days of the war, we were bombed all day long, which was extremely frightening. We stayed in the bomb shelter for two days, unable to get out! There were sirens, rocket attacks and bombing all day long. It was a nightmare; those were days of fear and helplessness*.

(P3)

The sense of threat was often intensified by the participants’ status as single parents. Most participants (N = 8) emphasized that the absence of a partner increased their own and their children’s vulnerability to becoming victims of terror attacks and being murdered by terrorists.

*I am scared of an invasion similar to the one that occurred in the southern region of the country. In the south, in most cases, the husband held the door of the bomb shelter against the terrorists while the mother stayed with the children, caring for them. In my case, as a single parent, I would be completely alone, totally exposed, and vulnerable to terror attacks and possible murder, along with my daughter*.

(P4)

Interestingly, most parents (N = 9) compared their fears stemming from the war to those arising from the COVID-19 pandemic, with some contending that their current death anxiety was absent during the pandemic.

*During the COVID-19 pandemic, I never thought that either I or my children might die. I had no medical risk factors, and they were young—young people don’t get severely affected by COVID. However, now I am terrified for our lives! I am truly frightened. We could actually die now. During COVID, I mostly worried about my parents, but I didn’t fear for my own life or my children’s lives the way I do now*.

(P5)

A major challenge described by all participants was the need to protect their children within the life-threatening context of the war, both physically and emotionally. Participants employed various protective practices tailored to the degree of life threat they faced. One such practice was using their own bodies to shield their children whenever sirens signaled incoming rocket attacks.

*I hold her in my arms during the sirens and missile attacks. I try to protect her with my body and calm her down*.

(P6)

One participant emphasized her increased efforts to save herself, knowing her survival was crucial as her children’s sole caregiver.

*Knowing that I am their only parent and that they have only me led me to concentrate on saving us. I knew that they need me and that they have only me, and it enhanced my efforts to save myself*.

(P1)

The majority of participants (N = 10) discussed efforts to protect their children’s psychological well-being from the war’s impact, using various practices. This included mediating the reality of war, a task they felt was especially important during wartime.

*Mediating such a complicated situation for children is incredibly hard. As parents, we consistently mediate reality to our children, even in times of peace. However, this task becomes much more complex during wartime. Yet, it remains one of our primary responsibilities as parents, and we must deal with it*.

(P7)

Several parents (N = 4) stressed the challenging task of mediating the situation in the context of lone parenthood.

*I have to support and explain the situation to them alone. I don’t have a partner to provide a second opinion or discuss ways to mitigate it for them. This is really tough*!

(P9)

Providing enhanced emotional support emerged as an additional parental practice for many (N = 7) during wartime. These parents noted that the psychological toll of the war on their children intensified their need for parental support.

*He is very anxious and frightened and cannot sleep at night. Therefore, I try to be supportive and caring as much as I can. I constantly try to calm him down and alleviate his fears. I stay physically close to him and haven’t been separated from him for a minute since the war started*!

(P8)

### 3.3. Financial Insecurity

Financial insecurity was perceived by many participants (N = 6) as another significant challenge of single parenthood in the context of the war. They emphasized their heightened vulnerability to war-related job loss and socio-political instability, as well as their expectation that institutional support would recognize and address their vulnerability to the adverse effects of financial instability during wartime.

*There are single mothers who lost their jobs during the war, and no one cares about them or their children. The National Insurance or the welfare system does not help them. How can they function as mothers if they have no money to provide for their children’s basic needs*?!

(P8)

In addition to individual financial struggles, many participants (N = 8) expressed concern about the war’s broader economic repercussions and the potential for national economic instability, emphasizing their heightened vulnerability as single parents.

*I am frightened, really frightened. If the war continues, the country’s economy will be destroyed. Fortunately, I have savings, so I can afford not to work for a while, but other single fathers and mothers I know are collapsing financially. Single parents are more vulnerable to national economic strains because they depend entirely on a single income and have no one else to provide for*.

(P10)

Similarly, most participants (N = 8) expressed a strong desire for a partner to share both the financial and emotional burdens of economic insecurity.

*I wish I had a partner with whom I could share my experience and who could support me. If something happens to me and I cannot work, nobody will help me—I am on my own! This feeling is more intense in times of crisis, like the current war, when I just wish I had someone, a partner, to share my emotional and financial burden*.

(P10)

Economic insecurity, as perceived by most participants (N = 8), was tied to political instability. This led some participants (N = 6) to express fears about the political repercussions of the war, which they believed could foster personal insecurity and even pose a threat to their safety.

*I am afraid that the war will lead to fascism and end our fragile democracy. In such a case, I, as a gay man and a single father, would become an easy target for political persecution, assaults, and even violence. This situation frightens me as much as the armed conflict itself*.

(P9)

### 3.4. Disruption to Daily Life

A key challenge that emerged from the interviews was the war’s disruption to the daily lives of the parents. All parents emphasized their struggles with basic routine tasks. Those who were evacuated from their homes experienced the greatest disruption to their routines.

*We now live in a hotel without any privacy. We lack autonomy over our daily schedule- we have to eat in the dining room with everybody else and not necessarily the food we like to eat. We all share a single room with our dog, it’s very intense and overcrowded. The kids are out of educational systems and with me all the time, so I can’t do anything*.

(P2)

All participants noted that their single status amplified the disruption to their daily routines.

*Most of my current hardships stem from my status as a single parent with no one to share the daily tasks. If I had a partner to help with activities like shopping, driving them to school, cleaning, and cooking, I would feel completely different. It’s always challenging to handle everything alone, but the war makes it much harder*.

(P5)

The disruption to daily routines was especially pronounced for parents of infants and young children (N = 3), as reflected in the words of a mother of a 15-month-old daughter.

*I can’t take a shower. I can’t go shopping. I can’t do anything because I can’t leave her even for a minute! Overall, I am totally on my own, and sometimes it feels like a nightmare*.

(P6)

Most parents (N = 8) noted that the difficulty of managing their daily routines alone evoked a desire for a partner with whom they could share their parental responsibilities.

*With all the responsibility for my children’s lives in this life-threatening situation, I wish I had a partner with whom I could share my obligations and daily tasks. Being a single parent during a war is extremely lonely and demanding, and sometimes it even feels like a nightmare*.

(P9)

### 3.5. Facilitating Factors in Parents’ Coping with War-Related Challenges

Participants’ accounts revealed three factors that facilitated their coping with the war-related challenges: joint familial activities, a supportive workplace, and social and political engagement. These factors fostered hope, contributing to their sense of empowerment and enhanced well-being.

### 3.6. Joint Familial Activities

The interviews demonstrate that the war context prompted many parents (N = 7) to engage more frequently in joint familial activities with their children. These parents described their increased participation in activities such as cooking, shopping, watching TV, and dining with their children as key sources of stress relief, positively impacting their well-being.

*I now try to ensure we have at least one shared meal every day. For dinner, and sometimes lunch, we cook and eat together. In the evening, we also watch TV together. These activities reduce my fears and improve my feelings*.

(P9)

Another positive impact of the increased joint activities with their children, as mentioned by parents, was the strengthening of their relationships and sense of belonging to each other. They emphasized the important role these strengthened bonds played in their own and their children’s survival within the war context.

*It is the two of us against the whole world. Our strong relationship, which has become even stronger during the war, is a shield that helps us survive this terrible situation. Undoubtedly, being together all the time under life-threatening attacks has brought us closer*.

(P4)

*Our joint activities increased our sense of belonging to each other. They enhanced our familial solidarity. They have had a profound impact on us, and I plan to make these activities a routine, even in ordinary times, after the war ends*.

(P11)

One father also noted the sense of meaning derived from his relationship with his son in the context of war.

*I am so glad that I have him, and that he has me. Knowing that we have each other helps me find meaning in all this mess and gives me a reason to struggle and survive these terrible times*.

(P10)

### 3.7. A Supportive Workplace

A supportive workplace was seen by all participants as essential for their coping during the war. Parents who worked in a workplace with a flexible work policy that enabled remote work and permitted absences during crises (N = 7) described fewer experiences of anxiety, depression, and sleep difficulties. They emphasized the major contribution of such supportive policies to their overall well-being.

*I was lucky that my workplace was very supportive and flexible. They saved my position, paid me a full salary, and even covered the cost of a substitute. I was really lucky! It gave me a sense of relief to know that at least I didn’t have to worry about my job*.

(P1)

A father of sixteen-year-old twin sons who was allowed to work from home, noted that this policy helped alleviate the strain of balancing his work and caregiving responsibilities.

*Ever since the war started, I have been working exclusively from home, and it has been very helpful because I can stay close to my sons. My job has a parent-oriented policy, which has been a great relief. I don’t have to juggle work and home responsibilities in such a stressful situation, and it really helps me navigate this crisis more easily*.

(P5)

Some (N = 6) emphasized that supportive work policies are particularly important for single parents, as they ease financial strain, allowing them to be more attuned to their children’s lives—a key benefit for sole caregivers.

*As a single father, working in a supportive and flexible workplace is especially helpful, particularly in times like these. At least I don’t have to worry about job security or financial difficulties. As the sole provider, this gives me a sense of security and reduces my stress, enabling me to be more attentive to my children*.

(P11)

Several (N = 4) parents emphasized the need for policymakers to recognize the heightened vulnerability of single parents to existing job market policies that do not accommodate their needs.

*I see other single parents without a supportive workplace. Some have been fired and have no one to support their children. I expect policymakers to consider the challenges faced by these parents and adopt job market policies that better address their needs*.

(P5)

### 3.8. Social and Political Engagement

Participants described involvement in two major types of activities that helped them cope with war-related challenges: volunteering and political activism. Both were perceived as sources of empowerment and enhanced competence.

Volunteering. All parents were engaged in volunteering activities within NGOs or their own communities. Many acknowledged the positive impact of volunteering on their well-being, with key benefits including its role in meaning-making and its contribution to an enhanced sense of agency and self-worth.

*The ability to contribute to others reduces the helplessness I feel, reduces some of the anger I feel and helps me feel valuable*.

(P7)

*Volunteering gave me a new meaning. I finally felt that I do something important for others and so did my children*.

(P5)

A mother of two sons, who had been evacuated from her home following the 7 October Hamas terror attack, emphasized the empowerment she derived from volunteering to help others in her community:

*What really improved my feeling was volunteering with the elderly in our community. I stay in touch with all the elderly from our ‘Kibbutz’ who are now staying at the hotel. Instead of crying all day, I focus on doing something for others, and it improves my feeling. It gives me great strength to know that I can help and contribute to those in my community*.

(P2)

Political involvement. Several parents (N = 4) viewed political involvement as a means of coping with the war and its consequences, emphasizing the importance of advocating for political change that would benefit their children.

*I argue with people online in favor of a new government that would fundamentally change this society. Being able to argue and advocate for a political change strengthens me and reduces some of the helplessness I feel. I cry out loud that we should end the war and seek a political solution to this endless conflict. I do so because I want my children to have a better future*.

(P9)

A mother of a sixteen-year-old daughter emphasized her sense of obligation as a mother to be actively involved in promoting political change.

*Since I am a mother, I feel that I should struggle for a better future in this country. Before the war, I used to attend demonstrations, and when the number of sirens decreases, I intend to return to protesting for a better future for my daughter. A better future that will be achieved through political change*.

(P3)

The urge to advocate for political change and a better future, along with the strengthened resilience it fosters, is also reflected in the words of a father of 10-year-old twin sons.

*I think we have to struggle to change this government and fight for our democracy for our children’s future. Unless we do that, they will not be able to live here. The thought of struggling for a different agenda… for future resolution helps me survive and fight for the sake of my children*.

(P7)

## 4. Discussion

This study investigated the experiences of Israeli SPCs under actual war conditions. The findings highlight the specific challenges these parents faced at the onset of the current phase of the Israel-Hamas war, which began on 7 October 2023, as well as the factors that mediated their impact. Three major war-related challenges emerged in participants’ accounts: a sense of danger and threat to life, financial insecurity, and disruption to daily life. Viewing these challenges through the lens of the SPM, which stresses the importance of acknowledging the structural contexts of individuals’ lives—including their social statuses [2] when analyzing the stress process, enabled us to identify two contextual factors that shaped their war exposure: single-parent status and the absence of institutional support tailored to the needs of SPCs during wartime. Similar contextual factors emerged during another major community crisis, where single-parent status and the lack of tailored institutional support significantly influenced the parenting experiences of single fathers by choice during the COVID-19 pandemic [28].

Participants described intense fear and deep concern for their children’s physical and emotional well-being amid the ongoing missile attacks across the country and the devastating aftermath of the October 7 terror attack, experiences also documented among married parents [17]. Yet, in contrast to findings from a nationwide perspective study on the mental health outcomes of the 7 October attack, which found women more prone to PTSD, depression, and anxiety [35], our findings reveal similarly pronounced emotional distress among both fathers and mothers. This disparity may be attributed to the primary caregiving role held by both fathers and mothers in our study due to their single-parent status, a factor not considered in Levi-Belz et al.’s research [35]. The emotional strain routinely experienced by SPCs, who bear sole responsibility for their children [47], appears to have been further exacerbated by the war in the absence of a partner to share fears and parental responsibilities. Our findings, highlighting the distinctive influence of single parenthood on conflict-related parenting experiences, align with an earlier study of individuals in southern Israel exposed to chronic conflict-related attacks, showing that single parents are at risk for PTSD symptomatology and global distress [48]. Nevertheless, that study did not specify the marital status of the single parents (e.g., divorced, widowed, or never married). By focusing specifically on SPCs, our study extends this earlier work and demonstrates that SPCs, like other groups of single parents, also face heightened vulnerability under conditions of ongoing conflict, providing nuanced insights into the interplay of single parenthood and wartime stress.

The contextual impact of participants’ single-parent status was also reflected in their accounts of major disruptions to their daily routines. Both mothers and fathers emphasized the increased complexity of managing daily routines and fulfilling multiple roles of parenting without a partner in the context of war. Enhanced role strain has also been observed among single mothers by choice [27] and single fathers by choice [28,49] during the COVID-19 community crisis. Additionally, participants emphasized their increased vulnerability to financial insecurity as single parents under war conditions. Some expressed frustration over the lack of institutional support for single parents, emphasizing their expectation that such assistance would acknowledge and address their vulnerable status as sole providers. This finding, consistent with the SPM [3], illustrates the role played by macro-level factors in shaping the experiences of SPCs in the context of armed conflict.

Our findings, indicating that participants regarded three distinct systemic-level factors as facilitating their coping with war-related challenges, corroborate the view of resilience as a multisystemic process, wherein protective factors within relational, sociocultural, and ecological systems interact to promote well-being amid adversity [13]. These findings also align with the SPM, which emphasizes the role of stress mediators in shaping stress outcomes [3]. One of the facilitating factors identified in our study, related to the relational system, was the participants’ strengthened bonds with their children, fostered by increased involvement in joint activities. This finding supports previous reports suggesting that strong family relationships facilitate coping with conflict-related stressors [15]. The findings also corroborate research on single fatherhood during the COVID-19 crisis, which highlighted the positive impact of increased parent-child time on single fathers’ well-being [28].

A supportive workplace emerged as another key mediator of war-related stress for participants. This organizational-level factor facilitated their coping with the heightened role strain of single parenthood [27,49], which generates unique challenges in balancing work and family life [47], further exacerbated by the war. Indeed, supportive workplaces with flexible policies are beneficial for single parents navigating these challenges [50].

Volunteering and political activism were also described by participants as mediators of war-related stressors. Participants linked the mediating role of volunteering to feelings of empowerment, self-worth, and a sense of meaning. This aligns with documented psychological benefits of volunteering, such as enhanced empowerment, positive affect, and a sense of purpose [51], which have also been observed during times of military conflict [52]. Beyond volunteering, some parents also gained a heightened sense of empowerment through active engagement in promoting political change aimed at ending the war and strengthening democracy. Advocating for a better future for their children enabled these parents to reclaim a sense of control, reducing their feelings of helplessness. Activism has similarly been identified as a protective factor and a source of agency for Palestinian women in the Gaza Strip within the context of armed conflict [15], and for Israeli mothers who engaged in the ‘Four Mothers’ protest movement, advocating the withdrawal of Israel’s military forces from southern Lebanon in the 1990s [53].

A major limitation of this study is its relatively small and homogeneous sample, which consisted primarily of middle- to upper-class, secular, and educated Jewish single parents living in central Israel. Although this demographic profile aligns with the reported profile of Israeli SMCs [23,25] and gay fathers through surrogacy [24,54], future research with larger and more socio-demographically diverse samples, including variation in social class and ethnicity, is needed. Such research can shed light on how SPCs’ wartime experiences, including their stress responses and resilience, are shaped by the intersection of their social identity as single parents with additional social identities related to their social class and ethnicity, thereby providing a more comprehensive understanding of experiences across different subgroups of SPCs. A second limitation concerns the use of Zoom for data collection, which presents several challenges. These include technical difficulties that may disrupt connectivity or reduce video and audio quality, as well as limited ability to observe participants’ nonverbal cues and body language, since their image is often displayed only from the waist up [55,56]. Another limitation concerns self-selection bias, whereby participants who chose to participate in this study may not fully represent the broader population of SPCs.

Future studies would benefit from exploring the experiences of SPCs who chose single parenthood after a prior divorce or widowhood, as well as those who became single parents due to divorce or widowhood, in contexts of political violence. Their experiences may differ from those of SPCs who have never been married or coparented children, enhancing our understanding of the nuanced realities within the group of single parents. Research should also examine how proximity to areas directly affected by terrorist attacks shapes parental well-being and mental health. In addition, cross-national studies, as well as studies exploring the wartime experiences of single parents in Gaza, the West Bank, or other regional contexts, are recommended to advance our understanding of single parents’ experiences across different conflict zones and cultural contexts.

Despite the study’s limitations, our findings shed light on the parenting reality faced by SPCs during armed conflict, showing that while their mental health is undermined, as it is for married parents in diverse conflict zones [57], it is also affected by unique challenges that exacerbate their emotional distress. The findings further highlight the factors that mediate the impact of these distinct challenges. Aligning with the SPM, which acknowledges that various social statuses, including marital status, play crucial roles in parental stress [58], our findings reveal how single parenthood amplifies parental vulnerabilities in the context of armed conflict. These findings, along with previous studies on single parenthood during the COVID-19 pandemic [27,28,49], deepen our understanding of how single-parent status affects parenting experiences during major community crises. Additionally, the stress mediators identified in our study contribute to the growing body of research on parental resilience in armed conflict settings, offering valuable insights into the relational, workplace, and sociopolitical processes that support single parents under armed conflict conditions. In doing so, these findings enrich multisystemic resilience frameworks by demonstrating how protective factors across diverse systems interact to mediate the effects of conflict-related stressors on SPCs’ mental health.

## 5. Conclusions

To conclude, by highlighting the distinct stressors faced by SPCs in wartime and the factors mediating their impact on well-being, as demonstrated in Table 4, our findings extend the Stress Process Model (SPM) to conflict settings, illustrating how stressors and coping resources interact not only in routine life but also under acute, life-threatening conditions. The findings show that the intersection of armed conflict and sole parenting responsibilities heightens vulnerability, while revealing both the coping strategies parents actively employ—such as joint familial activities and social and political engagement—and the external resources they benefit from, such as supportive workplaces, which help them manage war-related stressors and mitigate their impact on well-being, thereby offering new theoretical insight into parental adaptation under extreme adversity. Beyond the particular case of SPCs, this contribution advances the understanding of the contextual impacts of single parenthood on parenting experiences during major community crises.

On a practical level, our findings, underscoring the profound impact of armed conflict on SPCs’ well-being and mental health, call on professionals working with parents in conflict zones to adopt a nuanced approach that recognizes the distinct challenges faced by parents across diverse family structures and their associated mental health vulnerabilities. Specifically, the findings stress the urgent need to integrate comprehensive mental health considerations into clinical and psychosocial interventions for this population. Armed conflict compounds the already significant challenges of single parenthood, creating a complex web of stressors. These include absence of co-parenting support, increased role strain, sole responsibility for ensuring children’s physical safety and emotional stability, and profound social isolation, all of which may elevate distress and increase the risk of adverse mental health outcomes. This calls on mental health policymakers to broaden their approach to families in conflict zones by addressing the unique psychosocial concerns of SPCs and guiding clinicians and social workers in tailoring interventions to their specific needs. Tailored interventions might include tele-counseling, enabling SPCs to access psychosocial support and guidance for helping their children cope with war-related distress from home or shelters, without arranging childcare or having to travel with their children during dangerous conditions. They may also involve social workers advocating for workplace policies that address both SPCs’ financial and emotional vulnerabilities. Workplace settings are valuable platforms for mental health support during wartime [59]. This highlights the potential benefits of social workers—both occupational social workers and those operating in the community—actively engaging employers and advocating for supportive policies for SPCs. These targeted workplace interventions may complement broader institutional and community-level support systems, which are essential for addressing the structural dimensions of SPCs’ mental health risks.

The intersection of single parenthood and exposure to conflict, along with their familial implications, creates a unique clinical profile that may not be fully addressed through standard trauma-informed interventions. This emphasizes the need for mental health professionals to look beyond trauma exposure alone and consider the chronic and situational stressors defining the experiences of SPCs in conflict zones. The cumulative impact of these stressors can erode psychological resilience over time, and without adequate support, SPCs may experience a growing psychological burden that could impair their ability to function effectively.

Furthermore, participants in our study stressed the vital need for institutional and community-level support to alleviate the heightened burdens of single parenthood in conflict-affected environments. Their accounts highlight the structural dimensions of mental health risk, emphasizing that without targeted policies, accessible services, and robust safety nets, individual clinical efforts may be insufficient. Integrating mental health services with material, logistical, and social support systems, such as social service agencies, resilience centers, NGOs supporting single parents, and workplace settings, could significantly reduce distress and foster recovery, resilience, and long-term psychological stability for SPCs and their families.

Finally, the findings of this study emphasize that supporting the mental health of SPCs and their families during armed conflict is not only a clinical priority but also a critical, structural, social, and policy-driven imperative. Mental health professionals must collaborate across systems to address the unique vulnerabilities of this population, while simultaneously mobilizing and strengthening their inherent resilience and coping capacities.

## Figures and Tables

**Table 1:** Participants’ demographic characteristics.

Participant Number	^1^ Perceived Economic State	Martial History	Occupation	Education	Number of Children/Ages	Gender	Age	Region of Residence
P1	Good	Single	Social-worker	Master’s degree	2 (6,8)	Woman	44	South
P2	Good	Single	Cook	Post-high school	2 (13,6)	Woman	46	South
P3	Average	Single	Unemployed	High school	1 (16)	Woman	53	Central
P4	Good	Single	Medical lab worker	Master’s degree	1 (17)	Woman	58	Central
P5	Very good	Single	Banker	Master’s degree	2 (16) twins	Man	54	Central
P6	Average	Single	Photographer	Post-high school	1 (15 months)	Woman	47	Central
P7	Good	Single	Clerk	High school	2 (10) twins	Man	46	Central
P8	Good	Single	Medical secretary	Bachelor’s degree	1 (5)	Woman	40	Central
P9	Very good	Single	Banker	Bachelor’s degree	5 (13, 12, 11 & 5) twins	Man	58	Central
P10	Good	Single	Teacher	Master’s degree	1 (6)	Man	47	Central
P11	Good	Single	Physiotherapist	Master’s degree	2 (10) twins	Man	48	Central

^1^ Participants’ economic state was self-evaluated using four categories: (1) below average; (2) average; (3) good; and (4) very good.

**Table 2:** Sample coding memo—‘Sense of danger and threat to life’.

Participant	Memo Except	Researcher Notes
P3 (3 January 2024)	She is experiencing extreme fear due to the wartime conditions, including ongoing sirens and rocket attacks, which compel her to remain in the shelter with her daughter. She emphasizes their inability to leave the shelter during the rocket attacks and repeatedly expresses feeling a constant threat to her own and her daughter’s lives under these conditions.	This illustrates how the current war generates an acute sense of danger and a persistent feeling of threat to life.
P6 (7 January 2024)	She describes experiencing intense fear and anxiety in response to the hazards of war. She worries constantly about her own life and, even more, about her daughter’s safety. These fears are heightened by her status as a single mother, as she dreads the possibility that her daughter would be left entirely alone if something were to happen to her. Her anxiety extends beyond concern for herself to persistent fears for her daughter’s survival. In response, she tries to protect her by staying physically close and holding her tightly when the bombing begins.	This reveals how a pervasive sense of danger and threat to life shapes her mothering experience and practices, generating profound death-related fears for both herself and her child.
P5 (21 January 2024)	He expresses severe fear for his own life and that of his children, emphasizing the possibility that they could die during the war. His concern for his children’s safety leads him to stay at home to remain close to and protect them. He compares the wartime situation to the COVID-19 pandemic, during which he felt relatively safe at home, emphasizing the lack of death anxiety during the pandemic.	In this case, it is evident that perceived threat to life dominates the participant’s war-related experiences. All participants, regardless of their region of residence, reported feeling both emotionally and physically endangered during the war, reflecting a pervasive sense of threat to life and death anxiety. Thus, the term ‘threat to life’ seems to be an appropriate representation of participants’ experiences.

**Table 3:** Summary of themes, subthemes and operational definitions.

Theme	Subthemes	Operational Definitions	Illustrative Quotes
Challenges encountered during the war	Sense of danger and threat to life	Participants’ emotional experiences and parental practices within the life-threatening context	…now I am terrified for our lives! I am truly frightened. We could actually die now. (P5)
			I hold her in my arms during the sirens and missile attacks. I try to protect her with my body and calm her down. (P6)
	Financial insecurity	Participants’ financial concerns and burdens	How can they [SMCs] function as mothers if they have no money to provide for their children’s basic needs?! (P8)
			If something happens to me and I cannot work, nobody will help me—I am on my own. (P10)
	Disruption to daily life	Challenges to participants’ everyday routines and activities due to the wartime circumstances	The kids are out of educational systems and with me all the time, so I can’t do anything. (P2)
			I can’t take a shower. I can’t go shopping. I can’t do anything because I can’t leave her [daughter] even for a minute. (P8)
Facilitating factors	Joint familial activities	Participants’ efforts to foster joint familial activities and their impacts in the wartime context	I now try to ensure we have at least one shared meal every day. For dinner, and sometimes lunch, we cook and eat together. In the evening, we also watch TV together. These activities reduce my fears and improve my feelings. (P9)
			Our joint activities increased our sense of belonging to each other. They enhanced our familial solidarity. (P11)
	A supportive workplace	Participants’ experiences of a supportive workplace environment and its impact on their well-being	It gave me a sense of relief to know that at least I didn’t have to worry about my job. (P1)
			At least I don’t have to worry about job security or financial difficulties. As the sole provider, this gives me a sense of security and reduces my stress. (P11)
	Social and political engagement	Participation in community volunteering and activities aimed at political change	What really improved my feeling was volunteering with the elderly in our community. (P2)
			The thought of struggling for a different agenda… for future resolution helps me survive and fight for the sake of my children. (P7)

**Table 4:** Conceptual mapping of SPCs’ wartime stressors, mediators, and outcomes.

Stressors	Mediators	Outcomes
Sense of danger and threat to life; financial insecurity; disruption to daily life	Joint familial activities; supportive workplace; social and political engagement	Relieved stress; strengthened relationships with children; enhanced sense of belonging; increased sense of meaning; reduced anxiety, depression, and sleep difficulties; enhanced sense of agency and self-worth

## Data Availability

The data presented in this study are available on request from the corresponding author due to ethical reasons.

## Appendix A. Interview Guide

*Opening question*

Could you please tell me about your experience as a single parent during this war?

*Main questions*

1. What major challenges have you been facing as a single parent during the war?
  Do you think these challenges differ from those encountered by parents in two-parent families? If so, in what ways?
2. How have you been coping with these challenges?

Can you describe specific strategies or actions you have taken?

What parenting practices have you adopted, if any, in response to these challenges?

3. What has been helping you cope with these challenges?
4. What resources have facilitated your coping during the war?
5. Have you received support in your parenting role during this time?
  From particular individuals, community members, or organizations?
  From formal support services (e.g., social services, psychological support, municipal aid)?
  How helpful was this support?
6. What suggestions would you provide to mental health professionals and policymakers to better support and enhance the resilience of single parents during armed conflicts?
